# Prognosis of asymptomatic versus symptomatic metastatic breast cancer: a multicenter retrospective study

**DOI:** 10.1038/s41598-022-18069-z

**Published:** 2022-08-18

**Authors:** Sayaka Kuba, Shigeto Maeda, Shigeki Minami, Hiroki Moriuchi, Aya Tanaka, Momoko Akashi, Michi Morita, Chika Sakimura, Masayuki Baba, Ryota Otsubo, Megumi Matsumoto, Kosho Yamanouchi, Hiroshi Yano, Kengo Kanetaka, Takeshi Nagayasu, Susumu Eguchi

**Affiliations:** 1 Department of Surgery, Nagasaki University Graduate School of Biomedical Sciences, 1-7-1 Sakamoto-machi, Nagasaki, 852-8501 Japan; 2 Department of Breast and Endocrine Surgery, National Hospital Organization Nagasaki Medical Center, Nagasaki, Japan; 3 Department of Breast and Endocrine Surgery, Nagasaki Harbor Medical Center, Nagasaki, Japan; 4 Department of Surgery, National Hospital Organization Saga Hospital, Saga, Japan; 5 Department of Surgical Oncology, Nagasaki University Graduate School of Biomedical Sciences, Nagasaki, Japan; 6 Department of Surgery, Nagasaki Prefecture Shimabara Hospital, Nagasaki, Japan; 7 Department of Breast Surgery, Sasebo City General Hospital, Nagasaki, Japan

**Keywords:** Cancer, Breast cancer

## Abstract

In Japan, asymptomatic metastatic breast cancer (MBC) is often detected using tumor markers or imaging tests. We aimed to investigate differences in clinicopathological features, prognosis, and treatment between asymptomatic and symptomatic MBCs. Patients with MBC were retrospectively divided into asymptomatic and symptomatic groups to compare their prognosis by breast cancer subtype: luminal, human epidermal growth factor receptor 2 positive, and triple negative. Of 204 patients with MBC (114 asymptomatic, 90 symptomatic), the symptomatic group had a higher frequency of multiple metastatic sites and TN subtype. All cohorts in the asymptomatic group tended to or had longer post-recurrence survival (PRS) than those in the symptomatic group. In contrast, all cohorts and TN patients in the asymptomatic group tended to have or had longer overall survival (OS) than those in the symptomatic group, although no significant difference was observed in the luminal and HER2 subtypes. In the multivariate analysis, TN, recurrence-free survival, multiple metastatic sites, and symptomatic MBC were independently predictive of PRS. Regarding the luminal subtype, the asymptomatic group had longer chemotherapy duration than the symptomatic group, with no significant difference in OS between the groups. Asymptomatic and symptomatic MBCs differ in terms of subtypes and prognosis, and whether they require different treatment strategies for each subtype warrants further investigation.

## Introduction

According to Cancer Statistics 2021 by the American Cancer Society, it is estimated that nearly 1.9 million new cases of cancer will be diagnosed in 2021^1^. Breast cancer (BC) is the most common female cancer, accounting for 30% of all cancers. The estimated number of BC cases in Japan for 2021 is predicted to exceed 94,400, which will account for the highest number of BC cases ever in Japan^2^. Many guidelines recommend regular follow-up visits and annual mammography following BC treatment with curative intent^3–5^. Follow-up and surveillance aim to manage the physical and psychological effects of diagnosis and treatment, maximize adherence to adjuvant therapy, augment psychosocial support, and identify and manage any “curable” locoregional recurrences and second primary BCs that might occur^4^. Therefore, most guidelines advise against intensive follow-ups such as routine radiologic or blood-based surveillance (except for mammography) after curative treatment of early BC^3–5^.

In contrast, asymptomatic metastatic BC (MBC) is often detected in communities in Japan, as intensive follow-up is possible at patient’s request under the National Insurance System for Health, and on imaging tests for cancer screening at institutions outside of patients’ regular place of consultation. It is unclear whether asymptomatic MBC is just detected earlier before it becomes symptomatic and whether these two forms of MBC have similar biology and prognosis. Further, whether asymptomatic and symptomatic MBC have similar treatment approaches needs to be elucidated. This study sought to determine whether a difference existed between symptomatic and asymptomatic MBC in terms of subtype and clinical course, and if changing the treatment strategy should be considered depending on whether a patient has asymptomatic or symptomatic MBC. In MBC, where patients’ preferences and maintenance of quality of life are deemed important, predicting prognosis is useful to inform shared decision-making between patients and their healthcare providers. In particular, luminal BC (hormone receptor positive/human epidermal growth factor receptor [HER]2 negative) can be treated with either endocrine therapy or chemotherapy, with chemotherapy being the treatment of choice in cases of life-threatening disease^6^; however, there are inconsistencies in determining what qualifies as life-threatening disease. In addition, even if the disease is not life-threatening, long-term endocrine therapy may ultimately fail, necessitating the use of chemotherapy.

This study aimed to determine whether differences in clinicopathological features, subtypes, prognosis, and treatment of luminal BC exist between asymptomatic and symptomatic MBCs.

## Methods

### Patients

This retrospective study was approved by Nagasaki University Hospital Clinical Research Ethics Committee (approval number, 21091304), and the requirement for informed consent was waived. This study was performed in accordance with the ethical standards as described in the 1964 Declaration of Helsinki and its later amendments.

**Figure 1:**
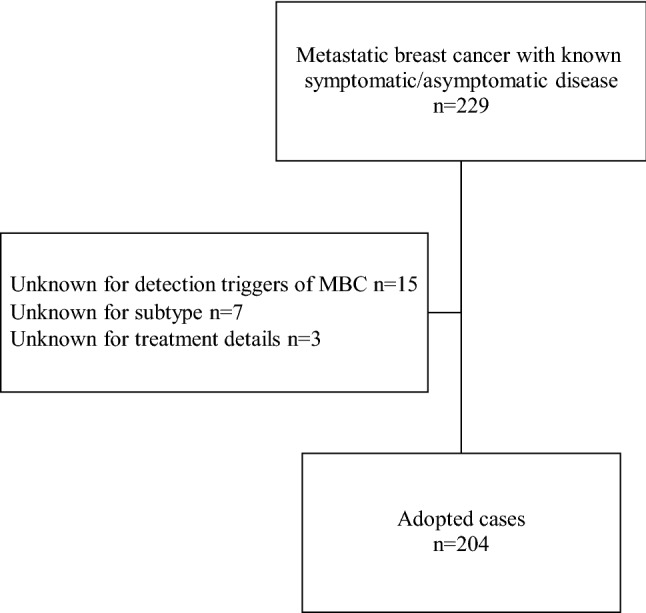
Flowchart showing the patient selection process.

We reviewed the medical records of patients with MBC who visited Nagasaki University, National Hospital Organization Nagasaki Medical Center, Nagasaki Harbor Medical Center, and National Hospital Organization Saga Hospital from 2008 to 2018. We defined MBC as the presence of organ metastasis or unresectable locoregional recurrence. We also excluded patients with de novo Stage IV BC and MBC whose symptomatic/asymptomatic status was unknown. Of the 229 patients with MBC with known symptomatic/asymptomatic status, 25 were excluded (15 for unknown detection trigger of MBC, 7 for unknown subtypes, and 3 for unknown treatment details) (Fig. 1). We did not perform any power analysis to determine the minimum number of participants to include in this study.

### Follow-up strategy and definition

The follow-up strategy was determined by the era of treatment, hospital, attending physicians, and patient preference; therefore, it was not uniform. The symptomatic group included patients who were aware of their symptoms or whose MBC was detected on examination by the physician without the use of imaging. The asymptomatic group included patients with MBCs detected on imaging and blood tests, including tumor markers (carcinoembryonic antigen and carbohydrate antigen 19-9), without symptoms. Among 204 patients with MBC, the BC subtypes were as follows: luminal BC (any hormone receptor positive/HER2 negative), HER2 BC (any hormone receptor/HER2 positive), and TNBC (triple negative BC; both of hormone receptor negative/HER2 negative). Patient age was defined as age at the time metastasis was confirmed. Recurrence-free survival (RFS) was defined as the time from the earliest treatment start date, either surgery or neoadjuvant treatment, to first recurrence. Overall survival (OS) was defined as the time from the earliest treatment start date, either surgery or neoadjuvant treatment, to death. Post-recurrence survival (PRS) was defined as the time from MBC diagnosis to death. Duration of endocrine therapy and chemotherapy was defined as the treatment period for MBC. Although rebiopsies of metastases sometimes showed different hormone receptor and HER2 expression from that at the initial surgery^7^, the subtype was determined by the status at the initial surgery. Multiple metastatic sites were defined not in terms of the number of metastases but as the presence of metastasis at multiple sites (e.g., bone and lymph nodes).

### Statistical analyses

Clinicopathological features were compared between the asymptomatic and symptomatic groups. Variables are expressed as frequencies for categorical variables and medians and ranges for quantitative variables. Associations between variables were assessed using Fisher’s exact test for categorical variables and the Mann–Whitney U test and Wilcoxon’s rank sum test for quantitative variables. Survival curves of PRS, OS, and durations of endocrine therapy and chemotherapy were constructed using the Kaplan–Meier method and compared using the log-rank test. Cox proportional hazards regression models were used to analyze PRS in RFS. Factors that were significant in the univariate analysis of survival were analyzed using a Cox proportional hazards regression model. All statistical analyses were performed using EZR software (http://www.jichi.ac.jp/saitama-sct/SaitamaHP.files/statmed.html). EZR is a graphical user interface for R (The R Foundation for Statistical Computing, version 2.13.0), which is a modified version of R Commander (version 1.6-3) that includes statistical functions frequently used in biostatistics^8^. Statistical significance was set at p < 0.05, and 0.05 ≤ p-value < 0.1 was considered a trend.

### Ethical approval

All procedures performed involving human participants were in accordance with the ethical standards of the institutional research committees and with the 1964 Helsinki declaration and its later amendments. The requirement for informed consent was waived.

## Results

### Relationship with clinicopathological factors between the symptomatic and asymptomatic groups

Of the 204 MBC patients, 114 were asymptomatic and 90 were symptomatic (Fig. 1, Table 1). The patients’ age at diagnosis of metastasis ranged from 26 to 91 years (median, 59 years). The BC subtypes were as follows: luminal, 120; HER2, 50; and TN, 34. The major detection triggers of MBC were locoregional mass palpation (43%), symptoms of bone metastasis (24%), and cough or dyspnea (11%) in symptomatic MBC and BC follow-up or screening imaging of other cancers (70%), and elevated tumor markers (29%) in the asymptomatic group. The symptomatic group had a higher frequency of multiple metastatic sites and chest wall recurrence than the asymptomatic group (Table 1). There was a significant difference between the subtypes in the presence or absence of symptoms; the luminal subtype was more prevalent in asymptomatic MBC, while the TN subtype was more prevalent in symptomatic MBC (p = 0.02, Table 1). Between the two groups, no significant difference was observed in age at the time of MBC diagnosis, RFS, presence or absence of visceral metastasis at the time of MBC diagnosis, or stage. When patients were divided by subtype, symptomatic MBC was associated with a higher frequency of chest wall recurrence (p = 0.006) and lesser frequency of liver metastasis (p = 0.05) than asymptomatic MBC in the luminal subtype. The symptomatic group tended to have a higher frequency of multiple metastatic sites than the asymptomatic group for the luminal (p = 0.06) and HER2 (p = 0.09) subtypes (Table 2).

**Table 1:** Comparison of clinicopathological factors between the asymptomatic and symptomatic groups (all cases).

	Asymptomatic MBC (n = 114)	Symptomatic MBC (n = 90)	p-value
Age^a^, years	59.5 (36–88)	59 (26–91)	0.93
Recurrence-free survival, months	40.5 (4–237)	41 (4–309)	0.99
Visceral metastasis^a^	70 (61.4%)	48 (53.3%)	0.32
Multiple metastatic sites^a^	40 (35.1%)	48 (53.3%)	0.01
**Metastasis site**^**b**^			
Lung	46	29	0.25
Bone	40	36	0.56
Lymph node	33	36	0.10
Liver	32	15	0.07
Chest wall	3	17	< 0.001
Brain	4	7	0.22
Stage 0/1/2/3	1/22/44/43/4	0/12/48/26/4	0.49
Subtype Luminal/HER2/Triple negative	75/27/12	45/23/22	0.02

*HER2* human epidermal growth factor receptor 2, *MBC* metastatic breast cancer.

^a^At the time of confirmation of metastasis

^b^There are duplicate cases.

**Table 2:** Comparison of clinicopathological factors between the asymptomatic and symptomatic groups according to subtype.

	Luminal		HER2		Triple negative	
	Asymptomatic MBC (n = 75)	Symptomatic MBC (n = 45)	Asymptomatic MBC (n = 27)	Symptomatic MBC (n = 23)	Asymptomatic MBC (n = 12)	Symptomatic MBC (n = 22)
Age^a^, years	62 (36–88)	59 (26–91)	56 (36–77)	56 (31–82)	56 (41–81)	59 (26–88)
Recurrence-free survival, months	46 (13–237)	54 (5–309)	54.5 (7–182)	54.5 (13–210)	20 (4–108)	22 (4–71)
Visceral metastasis^a^	49 (65.3%)	23 (51.1%)	16 (59.3%)	15 (65.2%)	5 (41.7%)	10 (45.5%)
Multiple metastatic sites^a^	28 (37.3%) ^⁋^	25 (55.6%) ^⁋^	8 (29.6%) ^⁋^	13 (56.5%) ^⁋^	4 (33.3%)	10 (45.5%)
**Metastasis site**^**b**^						
Lung	33	15	9	8	4	6
Bone	29	20	9	7	2	9
Lymph node	20	19	7	7	6	10
Liver	22^†^	6^†^	8	7	2	2
Chest wall	0^†^	5^†^	2	6	1	6
Brain	3	5	1	1	0	1
Stage 0/1/2/3/Unknown	0/14/30/28/3	0/4/28/12/1	1/6/8/11/1	0/4/7/9/2	0/2/6/4	0/4/13/5

*HER2* human epidermal growth factor receptor 2, *MBC* metastatic breast cancer, *OS* overall survival, *PRS* post-recurrence survival.

^a^At the time of confirmation of metastasis.

^b^There are duplicate cases.

^†^p-value less than 0.05.

^⁋^p-value greater than 0.05, less than 0.1.

### Comparison of PRS between the asymptomatic and symptomatic groups

**Figure 2:**
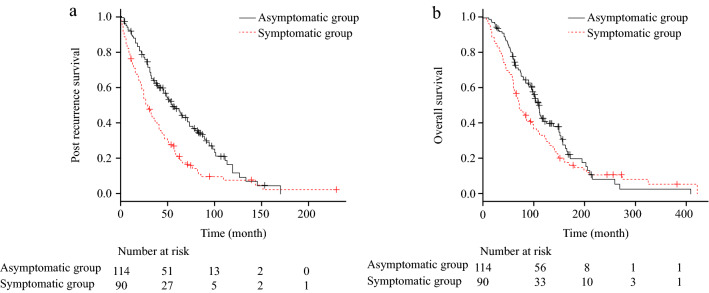
Survival analysis of all patients. (**a**) Post-recurrence survival (PRS), (**b**) overall survival (OS).

In all cases, PRS was significantly longer in the asymptomatic MBC than in the symptomatic group (median survival [95% confidence interval], 55 months [45–73] vs 29 months [22–39], respectively; p < 0.001) (Fig. 2a). In the TN subtype, PRS was significantly longer in the asymptomatic group than in the symptomatic group (28 months [8–100] vs 10.5 months [5–23], respectively; p = 0.01) (Table 3; Online Resource 1e). In the luminal group, PRS tended to be longer in asymptomatic MBC than in symptomatic MBC (54 months [46–73] vs 41 months [29–56], respectively; p = 0.08; Table 3; Online Resource 1a). In HER2, similar to the luminal group, PRS tended to be longer in asymptomatic MBC than in symptomatic MBC (71 months [25–89] vs 27 months [16–57], respectively; p = 0.09, Table 3; Online Resource 1c).

**Table 3:** Comparison of OS and PRS between the asymptomatic and symptomatic MBC groups according to subtype.

	Overall survival (months)	95% Confidence interval	p-value	Post-recurrence survival (months)	95% Confidence interval	p-value
**Luminal**						
Asymptomatic MBC	112	98–150	0.91	54	48–73	0.08
Symptomatic MBC	124	72–146		41	29–56	
**HER2**						
Asymptomatic MBC	113	59–195	0.40	71	25–89	0.09
Symptomatic MBC	72	49–112		27	16–57	
**Triple negative**						
Asymptomatic MBC	46	24–112	0.06	28	8–100	0.01
Symptomatic MBC	37.5	19–63		10.5	5–23	

*HER2* human epidermal growth factor receptor 2, *MBC* metastatic breast cancer, *OS* overall survival, *PRS* post-recurrence survival.

### Comparison of OS between the asymptomatic and symptomatic groups

In all cases, OS tended to be longer in the asymptomatic group than in the symptomatic group (110 months [96–126] vs 72 months [61–99], respectively; p = 0.09) (Fig. 2b). In TN, OS tended to be longer in the asymptomatic group than in the symptomatic group (46 months [24–112] vs 37.5 months [19–63], respectively; p = 0.06) (Table 3; Online Resource 1f). In patients with luminal and HER2 subtypes, OS was not significantly different between the two groups (Table 3; Online Resource 1b, d).

### Factors associated with PRS

PRS was associated with the TN/no TN subtypes (16 months [7–28] vs 49 months [40–58], respectively; p < 0.001), RFS (hazard ratio 0.927, 95% CI: 0.885–0.970; p < 0.001), and multiple/single metastatic sites (29 months [22–40] vs 50 months [39–64], respectively; p = 0.001) in univariate analysis. No significant difference in PRS was observed in the presence/absence of visceral metastasis at MBC diagnosis (p = 0.71) or among patients aged < or > 70 years (p = 0.55). Multivariate analysis of symptomatic/asymptomatic MBC, TN/no TN, RFS, and multiple/single metastatic sites revealed that these were independently predictive of PRS (Table 4).

**Table 4:** Multivariate analysis of post-recurrence survival.

	Hazard ratio	95% Confidence interval	p-value
**Subtype**			
Triple negative vs no triple negative	2.563	1.686–3.896	< 0.001
Recurrence-free survival, year	0.914	0.871–0.959	< 0.001
Multiple metastasis site vs single metastasis site	1.855	1.316–2.614	< 0.001
Symptomatic MBC vs Asymptomatic MBC	1.759	1.263–2.451	< 0.001

*MBC* metastatic breast cancer.

### Prognostic factors and treatment in luminal subtype

**Figure 3:**
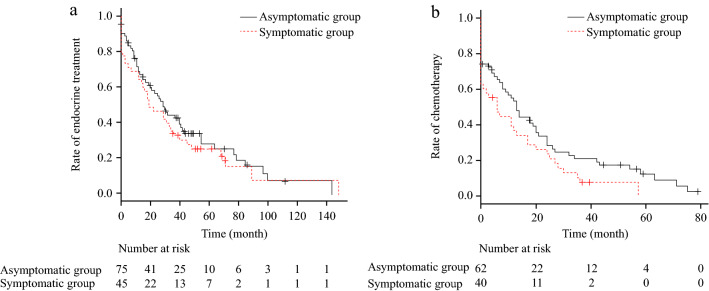
Treatment duration according to the asymptomatic/symptomatic luminal subtype. (**a**) Endocrine therapy, (**b**) chemotherapy.

Of the 120 patients with luminal BC, 101 underwent endocrine therapy. Of these, 11 were treated with cyclin-dependent kinase 4/6 (CDK4/6) inhibitors, 2 with m-TOR inhibitors, and 2 with sequential CDK4/6 inhibitors and m-TOR inhibitor; none used CDK4/6 inhibitors and m-TOR inhibitors as first-line treatment. In the luminal group, PRS was shorter in those with RFS of ≤ 4 years than in those with RFS of > 4 years (40 months [28–49] vs 66 months [49–83], respectively; p = 0.005). We divided the patients into two groups as follows: the group with predicted long-term PRS included patients who did not have any of the three risk factors (RFS of ≤ 4 years, multiple metastasis sites, and symptomatic MBC) and the group without predicted long-term PRS included those who had at least one of the three risk factors. The group with predicted long-term PRS comprised 21 patients (17.5%), and the group without predicted long-term PRS had 99 patients (82.5%); the PRS in the group with predicted long-term PRS was significantly longer than that in the group without predicted long-term PRS (80 [51–113] months vs. 41 [33–54] months, respectively, p = 0.008; Online Resource 2). Four of the 21 patients in the group with predicted long-term PRS were treated with concomitant CDK4/6 inhibitors. The duration of endocrine therapy did not differ between the asymptomatic and symptomatic groups (29 months [19–41] vs 19 months [12–34], respectively; p = 0.39, Fig. 3a; Online Resource 3). Regarding chemotherapy, of a total of 120 luminal patients, 71 underwent chemotherapy and 49 (18 survived and 31 died) did not undergo chemotherapy or discontinued chemotherapy after one cycle. Eighteen chemotherapy-naive and surviving patients were excluded because they may receive chemotherapy in future; in the remaining 102 patients, the duration of chemotherapy was longer in the asymptomatic group than in the symptomatic group (13 months [8–20] vs 6 months [0–13], respectively; p = 0.05 (Fig. 3b; Online Resource 3).

## Discussion

We compared asymptomatic and symptomatic MBC after curative treatment for BC based on four major findings. First, the proportion of subtypes differed significantly between patients with symptomatic and asymptomatic MBC. Second, in the luminal and HER2 BC subtypes, PRS tended to be higher in the asymptomatic group than in the symptomatic MBC group; conversely, no significant difference was observed in OS between both groups. Third, TN/no TN, RFS, multiple/single metastatic sites, and symptomatic/asymptomatic MBC were independently predictive of PRS. Fourth, the duration of endocrine therapy did not differ between asymptomatic and symptomatic MBC; in contrast, the duration of chemotherapy was longer in patients with asymptomatic MBC than in those with symptomatic MBC for the luminal subtype.

In our study, the luminal subtype was more prevalent in asymptomatic MBC, while the TN subtype was more prevalent in symptomatic MBC. One reason for this, regarding bone metastasis with the highest incidence in this study, may be that asymptomatic bone metastases were observed more in the luminal subtypes than in the TN subtypes. Our results are congruent with those of Di Gioia et al.^9^, who reported that among 29 patients diagnosed with MBC due to elevated levels of tumor markers, 72% had luminal subtype and 62% had bone metastasis. In contrast, Ogawa et al.^10^ conducted a retrospective study for intensive follow-up; no difference was observed between symptomatic/asymptomatic MBC and ER receptor and HER2 status, although the authors included both MBC and cancer with locoregional recurrence only.

We found no significant difference in OS between asymptomatic and symptomatic MBC in the luminal and HER2 subtypes. Since we had no provision for strict follow-up in this study, asymptomatic MBC and early detection of MBC are not synonymous; although asymptomatic, the early detection of MBCs would not have prolonged survival from the initial curative treatment. Our results are consistent with those of clinical trials showing that early detection of MBC by intensive follow-up does not prolong survival^11^. This result has also been reported in a recent retrospective study using an intensive follow-up of recent improvements in treatment and imaging^12^. Furthermore, early detection, such as the detection of circulating tumor DNA, was reported to be significantly associated with reduction in RFS in patients with early BC^13^; however, we should carefully judge whether early detection of the presence of circulating tumor DNA leads to prolonged OS. Furthermore, our results showed that the duration of chemotherapy was significantly longer in patients with asymptomatic MBC than in those with symptomatic MBC. Chemotherapy does not necessarily lead to a decrease in the patients’ quality of life; however, the impact of early detection on quality of life also needs to be investigated. In the TN subtype, PRS was significantly shorter, and OS tended to be shorter in the symptomatic group, suggesting that symptomatic MBC is predicted to be aggressive in nature. Our results revealed poor PRS compared with the nab-paclitaxel plus placebo group in the IMpassion130 trial of the first-line treatment of TNBC; however, we believe this was because patients who were ineligible for clinical trials were included^14^.

Predicting patients’ prognosis is useful for informing shared decision-making between patients and healthcare providers. We believe that it is important to discuss the importance of asymptomatic MBC as a prognostic factor with patients because asymptomatic MBC accounts for half of MBCs. Our results of a high proportion of asymptomatic MBC are consistent with Ogawa et al. who reported that 57% of patients were asymptomatic during an intensive follow-up cohort (including only those with local recurrence)^8^. Regarding the treatment strategy for the TN subtype, PRS was very short compared to that for other subtypes, especially in the symptomatic group. Moreover, palliative care should be introduced early in the diagnosis of MBC. Regarding treatment strategy for the luminal subtype, endocrine therapy combined with CDK4/6 inhibitors is the standard treatment^15^; however, it is not clear for which patients endocrine monotherapy would be an option for first-line treatment^16^. DeMichele et al.^17^ reported real-world evidence regarding the effectiveness of palbociclib plus letrozole versus letrozole alone in a large cohort of patients in routine clinical practice across the USA. They reported a median survival time of 43 months in a group of patients who used letrozole alone as first-line treatment, where 60% used CDK4/6 inhibitors as second-line or later treatment. In our study, most patients with asymptomatic MBC were not treated with CDK4/6 inhibitors; however, the median PRS of asymptomatic MBC was almost 1 year longer than that reported in the study by DeMichele et al.^17^. The median PRS of patients included in the group with predicted long-term PRS was 80 months, which was twice as long as that of those who received letrozole therapy alone as first-line treatment in DeMichele et al.’s report. Many patients who have received combined CDK4/6-endocrine therapy have been reported to convert to chemotherapy upon progression of MBC^18^. When patients with asymptomatic MBC are treated with this combination therapy as first-line treatment, they may convert to chemotherapy earlier without symptoms of MBCs and life-threatening disease. CDK4/6 inhibitors are used as second-line treatment, and they prolong survival; thus, patients defined as low-risk with long PRS in our study may be candidates for first-line hormonal monotherapy with fewer side effects and second-line CDK4/6 inhibitor therapy to ensure maximum survival time benefit.

This study has some limitations. First, the study population included patients diagnosed with MBC from 2015, and many cases did not undergo molecular targeted therapies, CDK4/6 inhibitors, trastuzumab emtansine (T-DM1), trastuzumab deruxtecan (T-DXd), or immune checkpoint inhibitors, which do not match the current treatment options. Second, although this was a multicenter retrospective study, the sample size was too small for analysis according to BC subtypes.

In conclusion, we demonstrated that asymptomatic MBC and symptomatic MBC differ in terms of subtype, prognosis, and duration of chemotherapy in the luminal group. Further studies are warranted to determine whether asymptomatic and symptomatic MBC require different treatment strategies according to subtype.

## Supplementary Information

Supplementary Legends.

Supplementary Information 2.

Supplementary Information 3.

Supplementary Information 4.

## Data Availability

The data that support the findings of this study are available from UMIN-CTR but restrictions apply to the availability of these data, which were used under license for the current study, and so are not publicly available. Data are however available from the authors upon reasonable request and with permission of UMIN-CTR.

## References

[1] American Cancer Society. Facts & figures. In *Reports Another Record-Breaking 1-Year Drop in Cancer Deaths*. https://www.cancer.org/latest-news/facts-and-figures-2021.html (2021).

[2] https://ganjoho.jp/reg_stat/statistics/stat/summary.html (2022).

[3] Runowicz CD (2016). American Cancer Society/American Society of Clinical Oncology breast cancer survivorship care guideline. J. Clin. Oncol..

[4] Cardoso F (2019). Early breast cancer: ESMO Clinical Practice Guidelines for diagnosis, treatment and follow-up. Ann. Oncol..

[5] NCCN clinical practice guidelines in oncology. In *Breast Cancer*. https://www.nccn.org/guidelines/guidelines-detail?category=1&id=1419 (2022).

[6] Hortobagyi GN (1998). Treatment of breast cancer. N. Engl. J. Med..

[7] Yamanouchi K, Kuba S, Eguchi S (2020). Hormone receptor, human epidermal growth factor receptor-2, and Ki-67 status in primary breast cancer and corresponding recurrences or synchronous axillary lymph node metastases. Surg. Today.

[8] Kanda Y (2013). Investigation of the freely available easy-to-use software ‘EZR’ for medical statistics. Bone Marrow Transplant..

[9] Di Gioia D (2015). Early detection of metastatic disease in asymptomatic breast cancer patients with whole-body imaging and defined tumour marker increase. Br. J. Cancer.

[10] Ogawa Y (2013). First indicators of relapse in breast cancer: Evaluation of the follow-up program at our hospital. Int. J. Clin. Oncol..

[11] Palli D (1999). Intensive vs clinical follow-up after treatment of primary breast cancer: 10-year update of a randomized trial. National Research Council Project on Breast Cancer Follow-up. JAMA.

[12] Cheun JH (2021). Intensity of metastasis screening and survival outcomes in patients with breast cancer. Sci. Rep..

[13] Cullinane C (2020). Association of circulating tumor DNA with disease-free survival in breast cancer: A systematic review and meta-analysis. JAMA Netw. Open.

[14] Schmid P (2018). Atezolizumab and Nab-paclitaxel in advanced triple-negative breast cancer. N. Engl. J. Med..

[15] Gennari A (2021). ESMO Clinical Practice Guideline for the diagnosis, staging and treatment of patients with metastatic breast cancer. Ann. Oncol..

[16] Cardoso F (2020). 5th ESO-ESMO international consensus guidelines for advanced breast cancer (ABC 5). Ann. Oncol..

[17] DeMichele A (2021). Comparative effectiveness of first-line palbociclib plus letrozole versus letrozole alone for HR+/HER2- metastatic breast cancer in US real-world clinical practice. Breast Cancer Res..

[18] Li Y (2021). A multicenter analysis of treatment patterns and clinical outcomes of subsequent therapies after progression on palbociclib in HR+/HER2- metastatic breast cancer. Ther. Adv. Med. Oncol..

