# Supplementary material for: Prognosis of asymptomatic versus symptomatic metastatic breast cancer: a multicenter retrospective study
Source: Sci Rep. 2022 Aug 18;12:14059. doi: 10.1038/s41598-022-18069-z (PMC9388511; doi:10.1038/s41598-022-18069-z)
Supplement: Supplementary file 1 — Supplementary Legends. [file 41598_2022_18069_MOESM1_ESM.docx]

**Online Resource 1.** Survival analysis according to subtype. (a) Post-recurrence survival (PRS) of luminal subtype, (b) overall survival (OS) of luminal subtype, (c) PRS of human epidermal growth factor receptor 2 (HER2), (d) OS of HER2, (e) PRS of TN (triple negative), (f) OS of TN

**Online Resource 2.** Survival analysis in luminal subtype with and without predicted long-term post-recurrence survival (PRS)

**Online Resource 3.** Time of endocrine therapy and chemotherapy in luminal subtype
