# Supplementary figures and images for: Prognosis of asymptomatic versus symptomatic metastatic breast cancer: a multicenter retrospective study
Source: Sci Rep. 2022 Aug 18;12:14059. doi: 10.1038/s41598-022-18069-z (PMC9388511; doi:10.1038/s41598-022-18069-z)

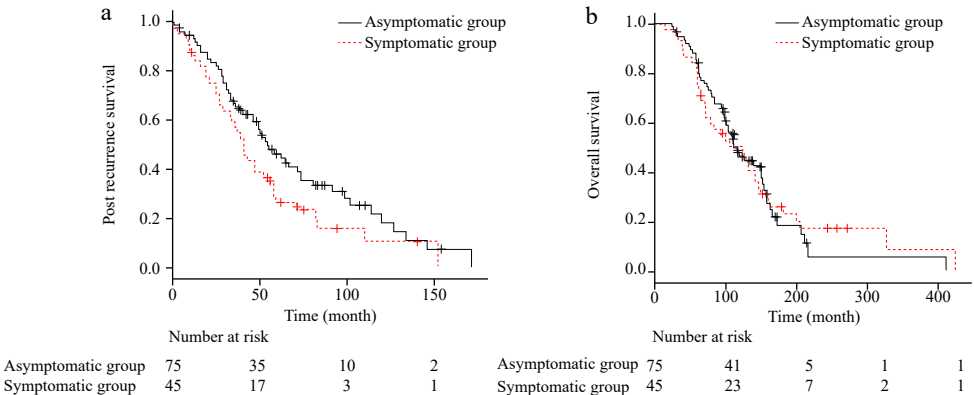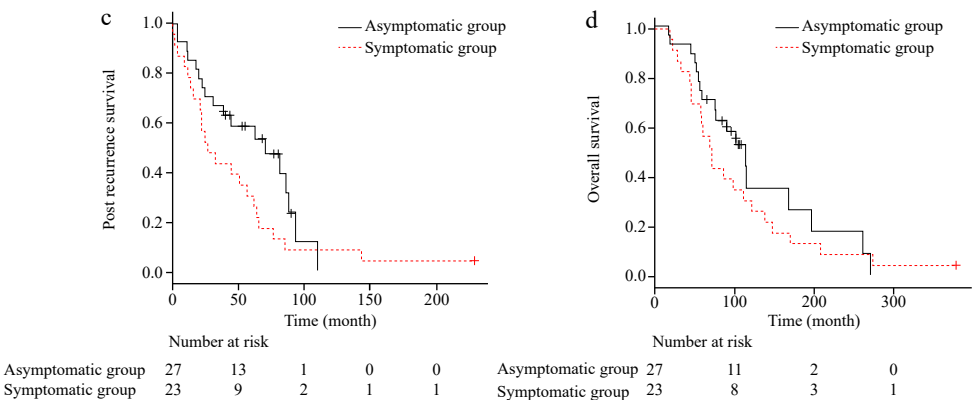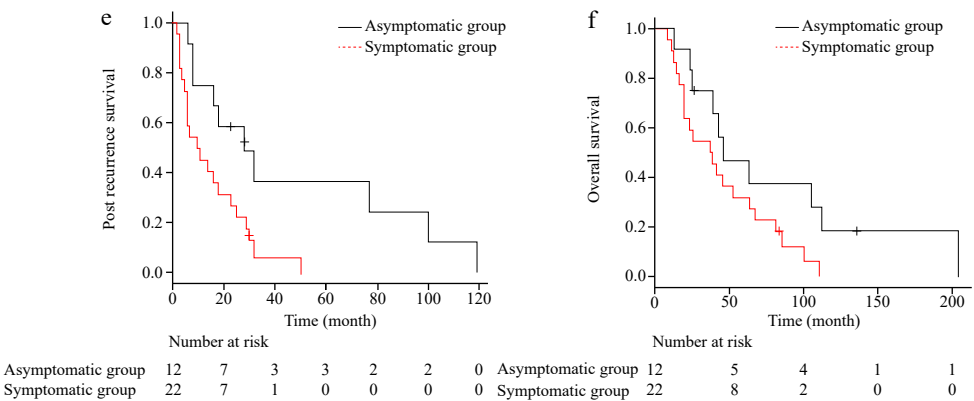

Supplement: Supplementary file 2 — Supplementary Information 2. [file 41598_2022_18069_MOESM2_ESM.pdf]

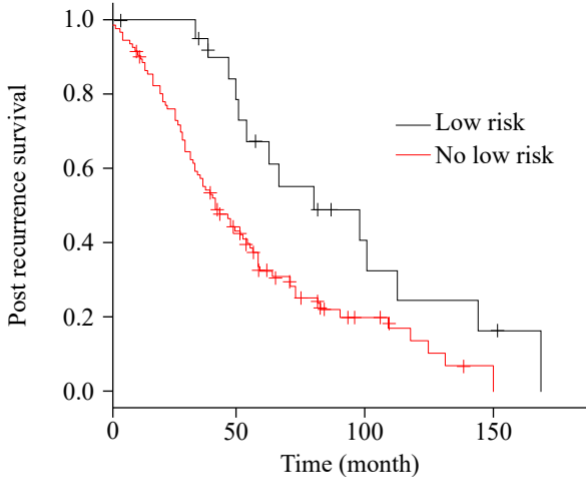

Number at risk

|             |    |    |   |   |
|-------------|----|----|---|---|
| Low risk    | 21 | 14 | 5 | 2 |
| No low risk | 99 | 38 | 8 | 1 |

Supplement: Supplementary file 3 — Supplementary Information 3. [file 41598_2022_18069_MOESM3_ESM.pdf]

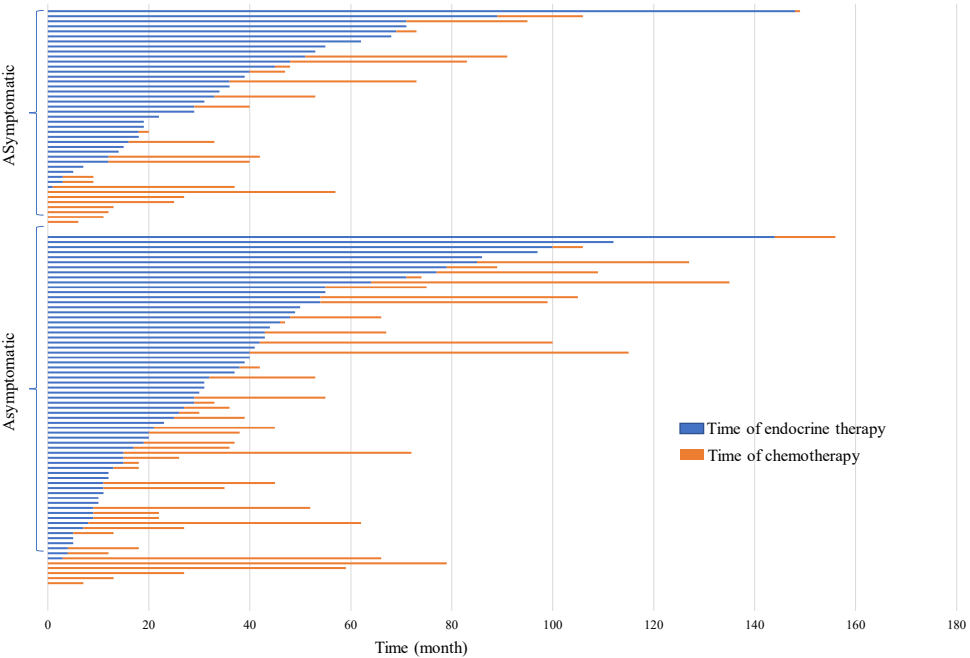

Supplement: Supplementary file 4 — Supplementary Information 4. [file 41598_2022_18069_MOESM4_ESM.pdf]
